# IL7 and IL7 Flt3L co-expressing CAR T cells improve therapeutic efficacy in mouse EGFRvIII heterogeneous glioblastoma

**DOI:** 10.3389/fimmu.2023.1085547

**Published:** 2023-02-03

**Authors:** Sheridan L. Swan, Nalini Mehta, Ekaterina Ilich, Steven H. Shen, Daniel S. Wilkinson, Alexa R. Anderson, Tatiana Segura, Luis Sanchez-Perez, John H. Sampson, Ravi V. Bellamkonda

**Affiliations:** ^1^ Department of Biomedical Engineering, Pratt School of Engineering, Duke University, Durham, NC, United States; ^2^ Duke Brain Tumor Immunotherapy Program, Department of Neurosurgery, Duke University Medical Center, Durham, NC, United States; ^3^ The Preston Robert Tisch Brain Tumor Center, Duke University Medical Center, Durham, NC, United States; ^4^ Department of Pathology, Duke University Medical Center, Durham, NC, United States; ^5^ Clinical Science Departments of Neurology and Dermatology, Duke University, Durham, NC, United States; ^6^ Department of Neurosurgery, Duke University Medical Center, Durham, NC, United States; ^7^ Department of Biology, Emory University, Atlanta, GA, United States; ^8^ Wallace H. Coulter Department of Biomedical Engineering, Emory University, Atlanta, GA, United States

**Keywords:** CAR T cells, glioblastoma, IL7, Flt3L, dendritic cells, T cell abundance

## Abstract

Chimeric antigen receptor (CAR) T cell therapy in glioblastoma faces many challenges including insufficient CAR T cell abundance and antigen-negative tumor cells evading targeting. Unfortunately, preclinical studies evaluating CAR T cells in glioblastoma focus on tumor models that express a single antigen, use immunocompromised animals, and/or pre-treat with lymphodepleting agents. While lymphodepletion enhances CAR T cell efficacy, it diminishes the endogenous immune system that has the potential for tumor eradication. Here, we engineered CAR T cells to express IL7 and/or Flt3L in 50% EGFRvIII-positive and -negative orthotopic tumors pre-conditioned with non-lymphodepleting irradiation. IL7 and IL7 Flt3L CAR T cells increased intratumoral CAR T cell abundance seven days after treatment. IL7 co-expression with Flt3L modestly increased conventional dendritic cells as well as the CD103+XCR1+ population known to have migratory and antigen cross-presenting capabilities. Treatment with IL7 or IL7 Flt3L CAR T cells improved overall survival to 67% and 50%, respectively, compared to 9% survival with conventional or Flt3L CAR T cells. We concluded that CAR T cells modified to express IL7 enhanced CAR T cell abundance and improved overall survival in EGFRvIII heterogeneous tumors pre-conditioned with non-lymphodepleting irradiation. Potentially IL7 or IL7 Flt3L CAR T cells can provide new opportunities to combine CAR T cells with other immunotherapies for the treatment of glioblastoma.

## Introduction

Glioblastoma (GBM) is a highly aggressive cancer and the most common malignant brain tumor (1). While current treatments of resection, radiotherapy, and temozolomide (TMZ) have doubled two-year survival rates to 18%, median survival remains around 14 months (2, 3). Chimeric antigen receptor (CAR) T cell therapy has shown promise in B cell non-Hodgkin’s lymphoma patients with 49% complete remission (4). Unfortunately, CAR T cell therapy is less successful in GBM with an overall median survival ranging from 7 to 24 months in various clinical trials (5–7). We sought out to enhance the efficacy of CAR T cell therapy in GBM by improving intratumoral CAR T cell abundance and modulating host immune cells in tumors pre-conditioned with non-lymphodepleting irradiation.

Lymphodepleting pre-conditioning using irradiation and/or chemotherapy can improve CAR T cell abundance by killing tumor cells, reducing competition for IL7 and IL15, and decreasing regulatory T cells (8–11). CAR T cell abundance in the peripheral blood was enhanced one week and four weeks post-infusion in neuroblastoma patients using lymphodepleting pre-conditioning (12). Despite these benefits, lymphodepletion severely reduces the host immune system. New strategies are needed to enhance CAR T cell abundance while preserving key immune cells like dendritic cells (DCs). DCs are antigen-presenting cells that generate specific T cell responses to combat disease. Exposure to 4 Gy irradiation depleted mouse splenic DCs whereas 0.5 Gy preserved around half of host DCs (13). In patients, tumor infiltrating lymphocytes can recognize and kill autologous tumor cells (14–16). Thus, there is a balancing act between the positive anti-tumor effects of lymphodepletion and the preservation of immune cells to maximize immunotherapeutic potential.

Significant efforts have been made to modify CAR T cells to improve intratumoral abundance. Modification of CAR T cells with a constituently active IL7 receptor enhanced CAR T cell expansion and survival in metastatic neuroblastoma and xenograft mouse models (17). However, this method was limited to intrinsically increasing the abundance of CAR T cells without targeting neighboring immune cells. IL7 is an attractive candidate to enhance the anti-tumor response of CAR T cells due to several key impacts on T cell biology. IL7 signaling in T cells promotes survival, proliferation, and, in certain circumstances, increases memory T cell formation and T cell receptor repertoire diversity (18–21). In comparison to IL2, IL12, and IL15, IL7 has shown low toxicity at a range of doses in clinical trials (22–26). However, IL7 has a limited half-life, around 9 hours in clinical studies, although some modifications have expanded this to 63 hours (26, 27). To circumvent poor half-life and localize IL7 delivery, CAR T cells can be engineered to secrete IL7. CAR T cells co-expressing IL7 and CCL19 or CCL21 improved overall survival and memory T cell formation in cyclophosphamide pretreated mastocytoma and pancreatic models (28, 29). These cells showed a complete response in 4 of 7 patients with refractory lymphoma when combined with anti-PD1 treatment (30). In a B cell lymphoma model, IL7 expressing CAR T cells have been shown to be in a less differentiated state with enhanced persistence (31). In glioma models, the use of IL7 is beginning to be explored. Subcutaneous delivery of modified IL7 increased systemic cytotoxicity of CD8 T cells and improved survival with co-administration of irradiation and temozolomide (TMZ) (32, 33). This therapy is in an ongoing clinical trial (NCT03687957) of intramuscular injections of modified IL7 in gliomas with irradiation/TMZ administration. While there is limited research on local IL7 delivery in brain tumors, one study demonstrated that co-delivery of intravenous CAR T cells with intratumoral IL7-loaded oncolytic adenovirus increased survival in GBM xenografts (34). These CAR T cells showed improved abundance but also increased exhaustion *in vivo*.

Another challenge of CAR T cell therapy is antigen heterogeneity resulting in the immunological escape of antigen-negative tumor cells. The epidermal growth factor receptor variant III (EGFRvIII) mutation can be found in approximately 30 – 40% of GBMs at varying levels and locations within a single tumor (35–38). Patients with recurrent GBM treated with a single dose of EGFRvIII CAR T cells experienced EGFRvIII antigen loss (39). Therefore, it is critical to utilize preclinical models that capture antigen heterogeneity to understand clinical translation. One approach utilized synNotch CAR T cells that have multi-antigen circuits for priming and killing in patient-derived xenografts (40). SynNotch CAR T cells significantly increased survival compared to conventional CAR T cells. While this animal model best represents patient heterogeneity, the use of immunocompromised mice eliminated the investigation of the endogenous immune response. Another approach delivered CAR T cells with oncolytic virus in a syngeneic GBM model with 100% or 10% EGFRvIII positive tumors (41). CAR T cells pre-loaded with virus in combination with oncolytic virus demostrated better therapeutic outcomes in 100% EGFRvIII positive tumors whereas 10% EGFRvIII positive tumors had limited efficacy. Similarly, in another study, CAR T cells in combination with a CAR T cell-boosting vaccine showed reduced efficacy with increasing ratios of antigen-negative tumors (42). Thus, it is critical to assess CAR T cell therapies in antigen heterogeneous tumors.

Maintaining the endogenous immune system is critical for mounting a T cell mediated response in antigen heterogeneous tumors. CAR T cell therapy has been shown to have enhanced efficacy in immunocompetent mice compared to immunodeficient mice (43). Endogenous T cells harvested from CAR T cell treated mice demonstrated significant anti-tumor activity *in vitro* and *in vivo* (43). Thus, enhancing the endogenous immune response could improve CAR T cell efficacy. Fms-like tyrosine kinase receptor 3 ligand (Flt3L) is a cytokine and growth factor essential for DC differentiation, expansion, and survival (44, 45). cDC1 is a key DC subset, known for surface expression XCR1, that can promote an anti-tumor CD8 T cell response (46). Therefore, increasing intratumoral cDC1 populations can potentially improve existing immunotherapies. Injections of Flt3L significantly increased CD103+ DCs (migratory DCs) in B16-OVA tumors (47). Another study, engineered CAR T cells to secrete Flt3L in Her2 tumors (13). These Flt3L CAR T cells increased conventional DCs and, in conjunction with polyinosinic-polycytidylic acid (poly(I:C)) and anti-41BB, enhanced T cell receptor diversity and epitope spreading. While promising, there are limited studies of Flt3L in the brain. Intratumoral injection of adenoviruses mediating tumor killing and delivering Flt3L in a rat GBM model resulted in increased plasmacytoid DCs (pDCs) and 70% long term survival (48, 49). A phase I dose escalation of this therapy in the glioma resection cavity demonstrated safety and promising preliminary survival outcomes (NCT01811992) (50). As far as we are aware, the exploration of Flt3L expression in the context of CAR T cell therapy in GBM has not been investigated.

In this study, we explored the effects of intratumoral delivery of CAR T cells expressing IL7 and/or Flt3L in a syngeneic EGFRvIII heterogeneous GBM model. We utilized non-lymphodepleting pre-conditioning to balance preserving the endogenous immune system while retaining the benefits of irradiation. IL7 and IL7 Flt3L CAR T cells enhanced intratumoral CAR T cell abundance and IL7 co-expression with Flt3L increased CD103+XCR1+ dendritic cells. IL7 and IL7 Flt3L CAR T cells enhanced overall survival in mouse EGFRvIII antigen heterogeneous tumors.

## Materials and methods

### Cell lines and media

CT2A, CT2A-EGFRvIII, and HEK293 cells were graciously donated by Dr. John Sampson’s lab. CT2A and CT2A-EGFRvIII were modified to express GFP and/or luciferase by transducing lentivirus made by the Duke Viral Core Facility (Addgene #89608 and #105621). Tumor cell lines were referred to as CT2A-GFP-Luc or 2A and CT2A-EGFRvIII-Luc or vIII. The presence of human EGFRvIII on CT2A-EGFRvIII-Luc was confirmed by flow cytometry with anti-human EGFRvIII antibody [L8A4] (Kerafast) (data not shown). Tumor cell lines were cultured in complete DMEM (cDMEM): DMEM, 10% FBS, 1%: Pen/Strep, Non-Essential Amino Acids (NEAA), and L-Glutamine. HEK293 cells, unless otherwise noted, were cultured in D10 media: DMEM with 4.5 mg/ml glucose and 0.11 mg/ml pyruvate (Gibco) + 10% FBS. T cells were cultured in T cell media (TCM) with or without human IL2 (donated from Dr. Sampson’s lab): RPMI 1640 (w/L-glutamine and sodium pyruvate), 10% FBS, 1%: Pen/Strep, NEAA, sodium pyruvate, L-glutamine, and 0.1%: β2-mercaptoethanol (Thermo Fischer), and 50 mg/mL gentamycin (Sigma-Aldrich).

### Retroviral CAR T cell production

CAR T cells were made using a previously established protocol (11). The pMSGV plasmid backbone with MSCV promoter was used to make third generation CAR T cells. Plasmids contained the human 139 single-chain antibody variable fragment (scFv) specific for EGFRvIII (19) along with transmembrane mouse CD8 and intracellular mouse CD3z, CD28, and 4-1BB signaling domains to enhance proliferation and function (51). When appropriate, plasmids were further modified to express mouse IL7 and/or Flt3L using an N-terminal secretion signal and P2A and/or T2A cleavage peptides. To make retrovirus, HEK293 cells were plated on a poly-l-lysine (Millipore Sigma) coated petri dish in D10 media. The following day, fresh D10 media was added to HEK293 cells and transfected using the previously described helper plasmid, pCL-Eco (Addgene), and lipofectamine 2000 (Invitrogen) in OptiMEM (Gibco). Spleens from 6- to 12-week-old female C57B6/J mice (Jackson Labs) were made into a single cell suspension using a 70 µm filter and washed with TCM without IL2. Cells were resuspended in RBC lysis buffer (BD) for 2 minutes and quenched with TCM. Cells were resuspended at 2x10^6 cells/mL in TCM with 2 µg/mL Concanavalin A (Sigma-Aldrich) and 50 IU/mL IL2 and plated in 24 well plates. The next day, fresh TCM without IL2 was added to HEK293 cells and, separately, a 24 well plate was coated with 25 µm/mL RetroNectin (Takara Bio) overnight at 4C. Concanavalin A activated splenocytes were transduced at 1x10^6 cells/mL with viral supernatant and spun for 1.5 hours at 2000 RPM at 32C. Fresh TCM with IL2 was added to each well. CAR expression on cells was assessed through flow cytometry on day 4 using a tetrameric peptide recognizing the CAR, CD3 Pe/Cy7 (17A2 BioLegend #100220), and CD8 PE (53-6.7 BioLegend #100708) on the NovoCyte 2060 or Cytek NL-3000. CAR T cells were used for *in vivo* experiments on day 5.

### Tetrameric peptide construction

A fluorescently labeled peptide to identify CAR T cells specific to EGFRvIII was made similar to a previous study (11). A custom peptide conjugated with biotin (JPT Peptide Technologies) was added in a 10:1 molar ratio with streptavidin-Alexa 647 (Invitrogen) in PBS to make a 1 mg/mL peptide solution. After incubation for 1 hour at room temperature in the dark, the solution was diluted to 0.5 mg/mL in PBS and incubated for another 2 hours. The solution was further diluted to 0.1 mg/mL and passed through a 30 kDa MWCO polyethersulfone filter (Millipore Sigma) to remove the free conjugated peptide. The tetrameric peptide was aliquoted and stored in the dark at 4C until use.

### *In vitro* enzyme-linked immunosorbent assay

ELISAs determined protein secretion of CAR T cells cultured alone or with tumor cells. 1x10^4 tumor cells in 200 µL of cDMEM were allowed to adhere for 5 hours in a 96-well plate. For the vIII + 2A condition, tumor cells were mixed in a 1:1 ratio with 0.5x10^4 EGFRvIII-positive and 0.5x10^4 EGFRvIII-negative cells before plating. cDMEM was removed from tumor cells and 1x10^5 CAR T cells were plated in 200 µL TCM without IL2. After 24 hours, the supernatant was harvested and frozen at -80C until assayed with IL7, Flt3L, IFNγ, or Granzyme B ELISA kits (R&D Systems) using the Spectramax i3x plate reader.

### *In vitro* bioluminescence assay

We used the bioluminescent signal from tumor cells as a surrogate marker for the tumor killing ability of CAR T cells. 1x10^4 tumor cells in 200 µL cDMEM were plated for 5 hours in an opaque 96-well plate. cDMEM was removed and replaced with 1x10^5 CAR T cells in 200 µL TCM without IL2. After 24 or 72 hours, Xenolight-D-luciferin (Perkin Elmer) was added at 150 µg/mL and incubated for 5 min until acquiring the signal on a Spectramax i3x plate reader (24 hour data not shown).

### Cell proliferation assay

We assessed CAR T cell proliferation by co-culturing CAR T cells with EGFRvIII-positive tumor cells. 1x10^6 CT2A-EGFRvIII-Luc cells were allowed to adhere to a 24-well plate for 5 hours. Separately, CAR T cells were stained per the manufacturer’s protocol with CellTrace Far Red (Thermo Fischer) for 20 minutes at 37C. Tumor media was removed from tumor cells and 1x10^6 CAR T cells were plated in TCM without IL2. Cells were incubated at 37C for three days and processed for flow cytometry. Samples were stained using Live/Dead Fixable Green reagent (Thermo Fischer) and anti-mouse CD8a PE antibody (53-6.7 BioLegend #100708).

### Surgical procedure

6- to 12-week-old female C57B6/J mice (Jackson labs) were used in accordance with the approved Duke Institutional Animal Care and Use Committee (IACUC) protocol. Mice were given Buprenorphine SR at 1 mg/kg and anesthetized using 3 to 5% isoflurane or 90 mg/kg ketamine with 10 mg/kg xylazine. Mouse skin was shaved and cleaned thrice with chlorhexidine and 70% ethanol. A small incision exposed the cranium and 1 to 2 drops of 0.25% bupivacaine were placed on the skull. Using a stereotaxic microinjector, a Hamilton syringe with a 25-gauge needle injected either 55,000 CT-2A-EGFRvIII-Luc or 75,000 CT2A-GFP-Luc plus 75,000 CT2A-EGFRvIII-Luc cells 1.5 mm posterior and left of bregma and 4 mm deep in 5 µL of 4000 cP methylcellulose (Sigma-Aldrich) with DMEM/F-12 (Thermo Fischer), sodium bicarbonate (Sigma-Aldrich), and HEPES (Thermo Fischer). The injection site was sealed with bone wax and wound clips closed the incision. Animals were monitored every two days for signs of distress or illness. 5 days following tumor injection, animals were imaged with IVIS Kinetic (Caliper Life Sciences) using an injection of 0.2 µm filtered Xenolight D-luciferin (Perkin Elmer) at 150 mg/kg body weight. Animals were randomized into treatment groups based on tumor size to allow for an equal distribution in each group. Animals without tumors based on bioluminescent imaging were excluded. On the indicated day, animals were irradiated with 0.5 Gy or 5 Gy irradiation using a Cesium-irradiator. The following day, animals were injected with 2x10^6 CAR T cells in 24 µL PBS at 60 µL/min at the same injection location.

### *In vivo* flow cytometry

Animals were euthanized 14 days following tumor inoculation. The brain was harvested and processed for flow cytometry based on previous protocols (52, 53). Briefly, animals were perfused with cold HBSS without Ca/Mg and the tumor-bearing hemisphere was placed in a digestion buffer (TCM without IL2, 50 mg/mL DNase I grade II from bovine pancreas (Roche), and 20,000 units/mL collagenase type IV (Gibco)). The digested tissue suspension was passed through a 100 µm filter and incubated on a tube rotator at 37C for 30 minutes. The suspension was filtered using a 70 µm filter, spun down at 500g, and resuspended in a 25% Percoll density gradient (GE Healthcare). After centrifuging at 521g at 18C for 20 minutes, the suspension went through multiple washes, red blood cell lysis buffer, and cell counting. The cells were blocked in mouse fc-block (BD biosciences), and stained with live/dead stain and antibodies. The following are the antibodies with clone noted and reagents from BioLegend unless otherwise noted: Zombie Aqua fixable viability kit (#423101), CD45 BV711 (30-F11 #103147), CD3 APC/Cy7 (17A2 #100222), CD8 BV650 (53-6.7 #100742), CD4 PE/Cy5 (GK1.5 #100410), CAR tetramer Alexa 647, PD-1 BV421 (29F.1A12 #135221), TIM3 BV605 (RMT3-23 #119721), LAG3 PE (C9B7W #125208), CD25 PE/Cy7 (3C7 #101916), CD69 BV785 (H1.2F3 #104543), CD11b APC/Cy7 (M1/70 #101226), F4/80 BV421 (BM8 #123137), CD11c BV605 (N418 #117334), MHCII PE (M5/114.15.2 #107607), CD103 PE-CF594 (M290 BD Biosciences #565849), CD45R PE/Cy7 (RA3-6B2 #103222), XCR1 APC (ZET #148206), CD86 BV785 (GL-1 #105043), and CD40 PE/Cy5 (3/23 #124618). The stained cells remained at 4C until read immediately on the BD LSRFortessa X-20. Animal studies were replicated twice. Data was analyzed using FlowJo v10.7.2.

### Bulk RNA and TCR sequencing

RNA was isolated from the tumor-bearing hemisphere and extracted using the RNeasy Lipid Tissue Mini kit (Qiagen). RNA purity was determined using the NanodropOne (Invitrogen) along with integrity and concentration measured with a Fragment Analyzer. The bulk RNA sequencing library was prepared using polyA tail enrichment with the KAPA Stranded mRNA-Seq kit (Roche). Sequencing was performed on the NovaSeq (Illumina) with 151 paired-end reads. For TCR sequencing, RNA was further processed with the mouse T-cell Receptor Panel QIAseq Immune Repertoire RNA Library Kit (Qiagen) using unique molecular indices with gene specific primers. Samples were run on MiSeq Version 3 (Illumina) using 300 paired-end reads. TCRseq data was analyzed using the online Qiagen platform with the IMSEQ algorithm (54). RStudio (R 4.2.1) was utilized for data processing, visualization, and statistics. For bulk RNAseq data, quality control was performed using FastQC/MultiQC. Star Alignment was run using default parameters and soft clipping for the Illumina universal adapter sequence. FeatureCounts was used for expression quantification of alignment output. Downstream analysis of the FeatureCounts raw counts output matrix was performed using DESeq2 (version 1.36.0). For differential gene expression analysis, to find genetic differences from responders and non-responders determined by LUC counts, one sample from each group was excluded based on principal component analysis clustering. The alternative shrink estimator ashr with a benjamini-hochberg correction was used to control for false discovery rates (FDR) (55). Differential gene expression was determined using an FDR < 0.1. The CIBERSORTx tool was used to infer cell fractions based on RNA sequencing data using the wild type samples from Seurat objects created from GSE197879 with S-mode batch correction and 100 permutations (56-58). Kyoto Encyclopedia of Genes and Genomes (KEGG) pathway analysis was performed using fast gene set enrichment on genes with a non-adjusted p-value < 0.05 (59). Cytosig analysis was performed using mean-centered log transformed data (60).

### Statistical analysis

When applicable, data has been presented using the mean + or - SEM. ELISA data was normalized using 10^x before statistical analysis. When applicable, a one-way analysis of variance (ANOVA) with Tukey’s posthoc test was used for analysis. When noted in the figure legend, groups were compared within tumor types (vIII, vIII + 2A, or 2A). The Kaplan-Meier plot was used for the survival curve and significant differences were assessed using the log-rank Mantel-Cox test. For TCR sequencing diversity metrics, a Kruskal-Wallis test with Dunn’s multiple comparisons was used to determine significance. A p-value of < 0.05 was considered significant. * = denotes significance to all other groups in the comparison. ns = denotes not significant. Unless otherwise noted, all statistical analysis was conducted using Graphpad Prism version 9.0.2.

## Results

### Construction and validation of IL7 and IL7 Flt3L co-expressing CAR T cells

**Figure 1:**
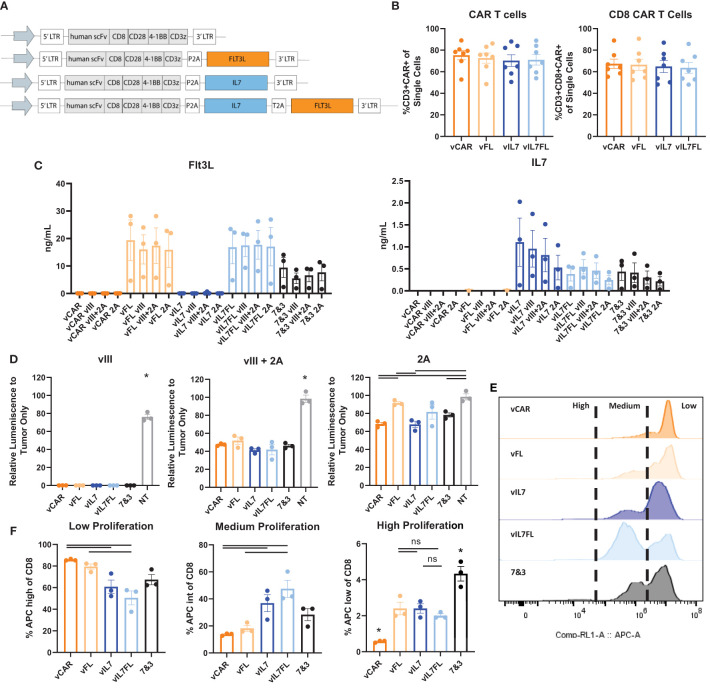
Construction and validation of IL7 and/or Flt3L CAR T cells. CAR T cells were made using activated mouse splenocytes and transduced on day 2. **(A)** Schematic of CAR T cell plasmids. **(B)** Flow cytometry of day 4 CAR T cells. Cells were stained with CD3, CD8, and a tetrameric peptide recognizing the CAR. **(C)** CAR T cells were cultured alone or co-cultured with tumor cells (vIII, vIII+2A, or 2A) with an effector:target (E:T) ratio of 10:1. 24 hours later the supernatant was collected for a Flt3L or IL7 ELISA. **(D)** CAR T cells were co-cultured with tumor cells with an E:T ratio of 10:1. 72 hours later bioluminescence signal was measured using a plate reader and normalized to tumor only signal. NT = non-transduced. **(E)** Cell proliferation of CAR T cells co-cultured with vIII tumor cells. CAR T cells were stained with CellTrace Far Red and plated at an E:T ratio of 1:1. After three days, cells were gated on live CD8 T cells and analyzed for APC expression indicating proliferation using a flow cytometer. Representative histograms of cell proliferation define low, medium, and high proliferation. **(F)** Quantification of cell proliferation from three replicates in one biological sample mean and SEM plotted. For all other experiments, each data point represents a biological replicate with mean and SEM plotted. Statistical analysis was conducted within each tumor group using a one-way ANOVA with a Tukey’s multiple comparison test. * = denotes significance compared to all other groups. ns, not significant.

Our first goal was to determine the functionality of IL7 Flt3L CAR T cells *in vitro*. We modified third generation CAR T cells by cloning mouse IL7 and/or Flt3L with an N-terminal secretion signal along with self-cleaving P2A and/or T2A peptides (**Figure 1A**). We confirmed no significant differences in the amount of CAR T cells from transduced splenocytes between CAR (vCAR), Flt3L (vFL), IL7 (vIL7), or IL7 Flt3L (vIL7FL) CAR T cells (**Figure 1B**). Additionally, cells were mostly CD8 CAR T cells due to the culture conditions. We then quantified secreted Flt3L and IL7 when CAR T cells were cultured alone or co-cultured with either CT2A-EGFRvIII-Luc (vIII), CT2A-GFP-Luc (2A) or 50% vIII and 50% 2A (vIII+2A) tumor cells. After 24 hours, we observed secretion of Flt3L and IL7 from CAR T cells programed to express those respective cytokines (**Figure 1C**). Interestingly, the expression of Flt3L remained stable despite the introduction of antigen-negative tumor cells, while IL7 expression seemed to diminish as 2A cells were introduced, although not significant. We further investigated delivering 50% vIL7 and 50% vFL, referred to as 7&3. While not significant, we did find lower levels of Flt3L for 7&3 compared to vFL and vIL7FL. We confirmed that CAR T cells secreted effector proteins through the expression of pro-inflammatory cytokine interferon gamma (IFNγ) and cytotoxic protein granzyme B (**Supplementary Figure 1**). Next, we measured tumor killing through a surrogate marker of bioluminescence signal due to luciferase expression in tumor cells. When CAR T cells were co-cultured with vIII the signal diminished compared to non-transduced splenocytes demonstrating effective elimination of tumor cells (**Figure 1D**). CAR T cells in the presence of vIII + 2A failed to eliminate luminescence signal. Notably, vCAR and vIL7 both significantly reduced luminescence signal compared to the non-transduced control when cultured with 2A for 72 hours. Since we confirmed IL7 secretion for CAR T cells, we assessed the functional effect of IL7 on T cell proliferation. CAR T cells were co-cultured with vIII at a 1:1 ratio and cellular proliferation was quantified after three days. Low proliferation was defined as the peak with the highest amount of signal, medium proliferation represented the intermediate population, and high proliferation reflected the population with the lowest signal (**Figure 1E**). In comparison to vCAR, vIL7 and vIL7FL showed a significant increase in the percentage of medium and high proliferating CD8 T cells (**Figure 1F**). Thus, IL7 secreted from vIL7 and vIL7FL had the capacity to increase proliferation *in vitro*.

### IL7 and IL7 Flt3L expressing CAR T cells increased CAR T cell abundance in 5 Gy TBI EGFRvIII positive tumors

**Figure 2:**
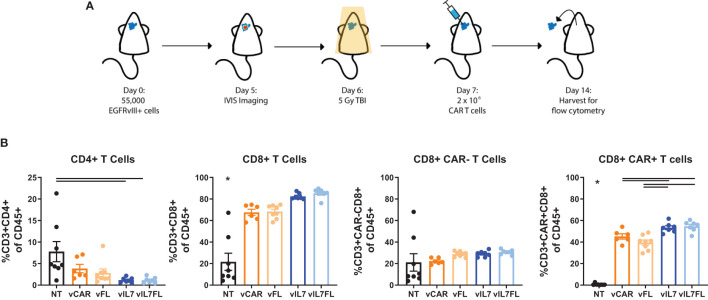
IL7 and IL7 Flt3L co-expressing CAR T cells increased the CD8 CAR T cell population in a 5 Gy TBI EGFRvIII homogenous model. **(A)** Experimental timeline. Animals were inoculated intracranially with 55,000 vIII tumor cells. Tumors were IVIS imaged five days later and subjected to 5 Gy irradiation on day 6. On day 7, 2x10^6 CAR T cells were injected intracranially. **(B)** The tumor was harvested for flow cytometry on day 14 and analyzed for T cell populations (n=6-8). A one-way ANOVA with a Tukey’s multiple comparison test determined significance. Mean and SEM are plotted. * = denotes significance compared to all other groups.

Since we observed increased proliferation of CAR T cells secreting IL7 *in vitro*, we assessed the intratumoral presence of CAR T cells 7 days after *in vivo* delivery. Previous studies utilizing TMZ for lymphodepleting pre-conditioning demonstrated peak CAR T cell abundance in the blood one week post-treatment and showed significant increases in intratumoral CAR T cells 7 days after treatment (11). 100% EGFRvIII positive tumors were inoculated into mice that received 5 Gy total body irradiation (TBI) the day before intracranial injection of CAR T cells (**Figure 2A**). One week after CAR T cell delivery, tumor tissue was processed for flow cytometry. vIL7 and vIL7FL significantly increased the CD8+ CAR T cell percentage of CD45+ cells compared to vCAR and vFL (**Figure 2B**). There was no change in CAR-negative (CAR-) T cells between groups. This is most likely due to the lymphodepletion caused by 5 Gy irradiation (13).

### IL7 Flt3L CAR T cells increased CAR T cell and dendritic cell populations in EGFRvIII heterogeneous tumors treated with non-lymphodepleting irradiation

**Figure 3:**
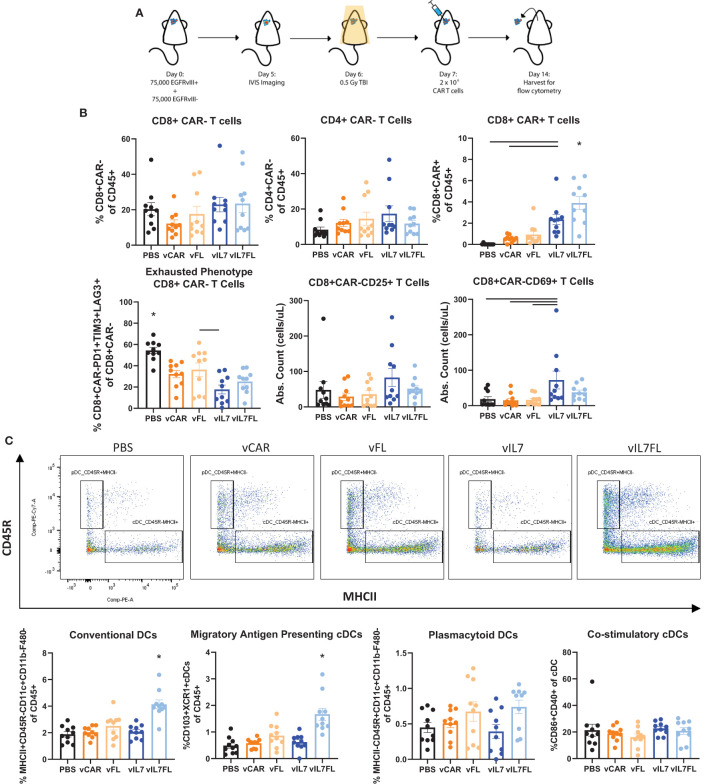
IL7 and IL7 Flt3L co-expressing CAR T cells increased CD8 CAR T cells and IL7 co-expression with Flt3L enhanced intratumoral DCs. **(A)** Schematic of experiment. Animals were inoculated with 50% vIII and 2A tumors and IVIS imaged 5 days later. On day 6, 0.5 Gy TBI was applied and 2x10^6 CAR T cells were injected intracranially the following day. **(B)** Flow cytometry was performed on the tumor-bearing hemisphere isolated on day 14 and split into two panels with the analysis of the T cell panel shown. **(C)** Analysis of DC populations using flow cytometry. Data represents a combination of two independent experiments (n=10). Statistical test was a one-way ANOVA with a Tukey’s multiple comparison test with mean and SEM plotted. * = denotes significance compared to all other groups.

Next, we examined the ability of vIL7FL to alter immune cell populations in a more rigorous animal model. Animals were inoculated with tumors containing 50% EGFRvIII positive and 50% EGFRvIII negative cells. Prior to CAR T cell injection, a non-lymphodepleting dose (0.5 Gy) of irradiation was administered (**Figure 3A**). Compared to 100% EGFRvIII syngeneic mouse tumors, this model better recapitulates antigen heterogeneity while preserving the endogenous immune system and the anti-tumor benefits of irradiation. Using a multi-color flow cytometry panel, the tumor-bearing hemisphere was analyzed one week after CAR T cell delivery for T cells and dendritic cells (DCs) (**Supplementary Figure 2**). vIL7 and vIL7FL significantly increased intratumoral CAR T cells compared to vCAR (**Figure 3B** and **Supplementary Figure 3**). Interestingly, vIL7FL had significantly higher CD8 CAR T cells compared to all groups, including vIL7. This could suggest a synergistic effect from IL7 and Flt3L. There was no significant difference in CAR- CD8 T cells. The introduction of any CAR T cell significantly reduced the proportion of PD1+TIM3+LAG3+ of CAR- CD8 T cells, referred to as phenotypically exhausted T cells, compared to PBS. Notably, vIL7 significantly decreased exhausted T cells compared to vFL. Additionally, we assessed activation of CAR- T cells through expression of CD25 or CD69. While the proportion of CD25+ or CD69+ T cells of CD8 CAR- T cells did not significantly differ, vIL7 significantly increased absolute counts of CD69+ CD8 CAR- T cells compared to PBS, vCAR, and vFL (**Supplementary Figure 3** and **Figure 3B**). Next, we investigated intratumoral conventional DCs (cDCs) due to their importance in antigen presentation. cDCs were classified by surface expression of CD45+F480-CD11c+CD11b-CD45R-MHCII+. vIL7FL enhanced intratumoral cDCs as well as the CD103+XCR1+ subset known as migratory antigen-presenting DCs (**Figure 3C**). There was no significant difference in plasmacytoid DCs.

### IL7 and IL7 Flt3L CAR T cells improved overall survival and altered gene expression in EGFRvIII heterogeneous tumors treated with non-lymphodepleting irradiation

**Figure 4:**
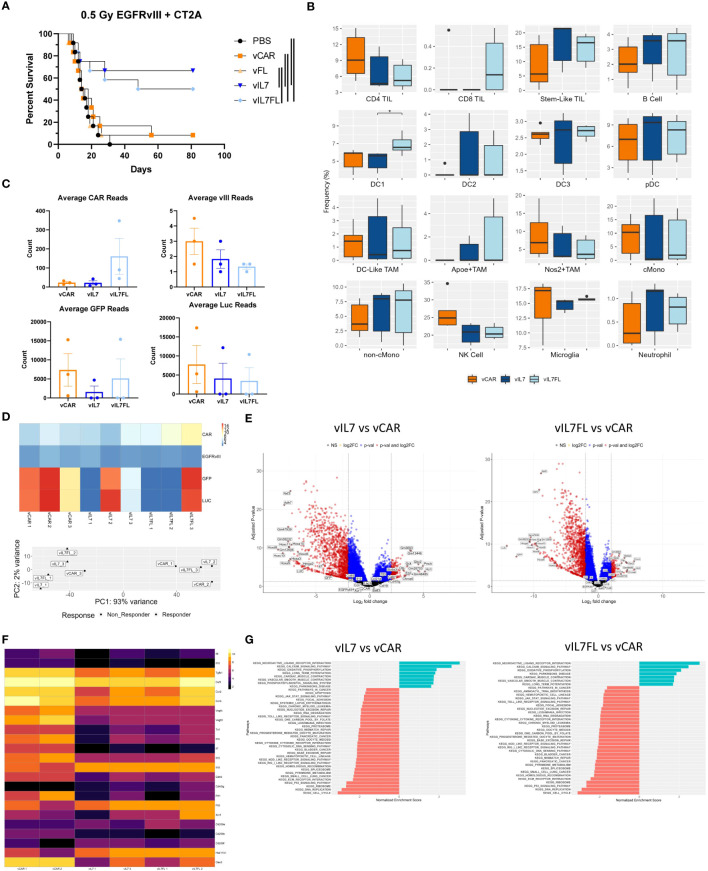
IL7 and IL7 Flt3L co-expressing CAR T cells increased survival in a 0.5 Gy TBI EGFRvIII heterogeneous model. Animals were inoculated with 50% vIII and 2A tumor cells and IVIS imaged 5 days later. **(A)** On day 6, 0.5 Gy TBI was applied and 2x10^6 CAR T cells were injected intracranially on day 7 and overall survival was assessed (n=12). Kaplan-Meier survival curves represent a combination of two independent experiments. Significance is noted with the log-rank Mantel-Cox test. **(B)** 7 days after tumor inoculation 0.5 Gy TBI was applied and 2x10^6 CAR T cells were injected intracranially on day 7. One week post CAR T cell injection, the tumor-bearing hemisphere was isolated for bulk RNA sequencing (n=3). **(C)** Transgene counts were found by averaging the lane reads in each sample. **(D)** Transgene heatmap using normalized data and principal component analysis. **(E)** Volcano plots with thresholds of log2 fold change = 2 and adjusted p-value = 0.05. **(F)** Normalized heatmap of selected differentially expressed genes. **(G)** KEGG analysis using non-adjusted p-value < 0.05 with an adjusted p-value of < 0.1 Differential expression between transgenes and all other genes was found using alternative shrink estimator ashr with a Benjamini-Hochberg correction with a FDR < 0.1.

We evaluated the therapeutic outcome of vIL7 and vIL7FL in EGFRvIII heterogeneous tumors treated with non-lymphodepleting irradiation. Animals were subjected to 0.5 Gy TBI 6 days after tumor inoculation with 50% EGFRvIII positive and 50% EGFRvIII negative tumor cells. On day 7, CAR T cells were delivered intracranially, and animals were monitored for survival. vIL7 and vIL7FL significantly enhanced survival compared to PBS, vCAR, and vFL (**Figure 4A**). Interestingly, vCAR and vFL had a survivor which could be due to the bystander effect from using irradiation (61). Bioluminescent imaging of tumors 5 days post-treatment indicated repression of tumor burden in vIL7 and vIL7FL (**Supplementary Figure 4A**). To understand the effect of vCAR, vIL7, or vIL7FL treatment on a transcriptional level, we evaluated tumor gene expression 7 days after CAR T cell delivery using bulk RNA sequencing. First, we assessed inferred cell fractions using CIBERSORTx. We utilized previously published single cell RNAseq data of mouse CD45 pre-sorted CT2A tumors to infer immune cell populations from bulk RNAseq data. The cDC1 population was significantly increased in vIL7FL compared to vIL7, which correlated with our flow cytometry data (**Figure 4B**). Since EGFRvIII positive tumor cells expressed EGFRvIII and luciferase (LUC) and EGFRvIII negative tumor cells expressed GFP and LUC, we used these transgenes as a surrogate markers for antigen-positive and negative tumor cells. GFP and LUC transgenes displayed a responder and non-responder effect (**Figure 4C**). The principal component analysis revealed that one sample from each group did not cluster with the other samples and samples were differentiated by LUC counts (**Figure 4D**). Additionally, the responder status did not necessarily indicate increased CAR reads (**Figure 4D**). Thus, to determine transcriptional differences between responders and non-responders to generate hypotheses for future studies we excluded the following samples that didn’t cluster: vCAR 3, vIL7 2, and vIL7FL 3. LUC and GFP were differentially expressed between vIL7 vs. vCAR and vIL7FL vs vCAR (**Figure 4E**). This was to be expected based on our exclusion criteria. There were no differentially expressed genes between vIL7 vs vIL7FL. vCAR treatment upregulated various differentially expressed immunosuppressive and immunostimulatory cytokines, including IL7 (**Figure 4F**). KEGG pathway analysis found enrichment in the neuroactive ligand receptor interaction pathway (**Figure 4G**). Upregulation of this pathway has been associated with glioblastoma (62, 63). However, one study found GBM patients with deficiencies in the neuroligand receptor interaction pathway have a poor prognosis due to mutations or low expression of Calcr (64). vIL7 and vIL7FL increased differential gene expression of Calcr compared to vCAR. However, the role of Calcr in GBM remains ambiguous. One common adverse event in CAR T cell therapy is cytokine release syndrome (CRS). CRS can occur 1 to 14 days post-CAR T cell therapy resulting in elevated levels of cytokines, including IL2, IL6, IL10, and TNF (65, 66). We did not observe overt weight loss 14 or 15 days post CAR T cell delivery in either experimental cohort (**Supplementary Figure 4B**). Additionally, the Cytosig platform was used to predict cytokine signaling in tumors 7 days post CAR T cell injection using gene expression data from bulk RNA sequencing (60). While there was a slight upregulation of IL6 and CD40L, which is known to induce IL6, TNF and IL2 were down-regulated in the treatment responders (**Supplementary Figure 4C**) (67, 68).

### IL7 and IL7 Flt3L CAR T cells effect on T cell receptor repertoire diversity

**Figure 5:**
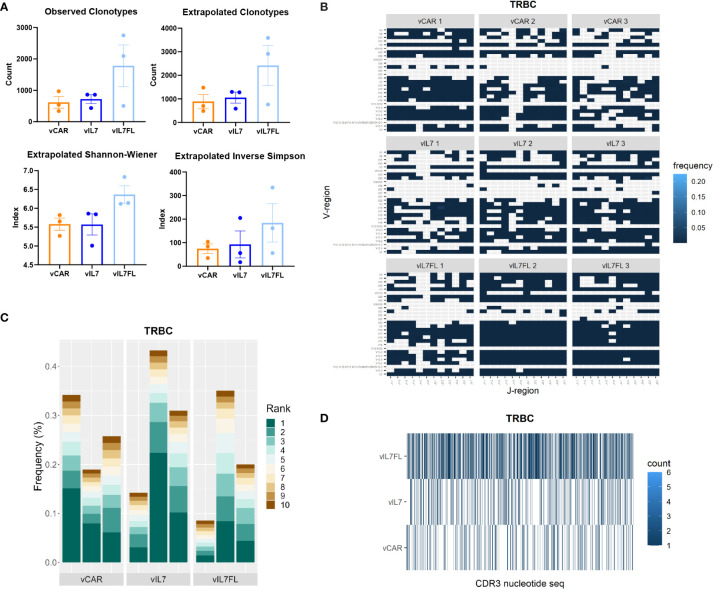
TRBC diversity of IL7 Flt3L co-expressing CAR T cells. Animals were inoculated with 50% vIII and 2A tumor cells and IVIS imaged 5 days later. On day 7, 0.5 Gy TBI was applied and 2x10^6 CAR T cells were injected intracranially the following day. RNA was extracted from the tumor-bearing hemisphere 7 days post CAR T cell injection and processed for immune repertoire sequencing (n = 3). **(A)** Diversity metrics of TRBC. Statistical analysis was a Kruskal-Wallis test with Dunn’s multiple comparisons. **(B)** Heat map of V and J pairings of TRBC. **(C)** The frequency of the top 10 clonotypes of TRBC. **(D)** Visualization of TRBC clonotypes combing biological replicates.

Finally, we investigated the impact of vIL7FL on T cell receptor beta chain (TRBC) diversity. We assessed the T cell receptor (TCR) alpha and beta chains due to their importance in immunotherapy (69). The alpha and beta protein chains are created through recombination of variable (V), joining (J) and, in certain cases, diversity (D) gene segments. The alpha chain utilizes V-J recombination, while the beta chain uses V-(D)J recombination. While both are required together for antigen recognition, the TRBC is recognized as more uniquely expressed in T cells than the alpha chain, due to the additional D recombination and allelic exclusion, and therefore is the focus of this study (70–72). First, we compared the observed number of clonotypes, and asymptotic diversity metrics found by extrapolating the unique molecular indices to infinity (**Figure 5A**). The asymptotic number of clonotypes was found using chao 1 statistics. While not significant, vIL7FL tended to have more clonotypes and higher indexes of diversity. We then visualized V-J gene segment pairings between groups. vIL7FL qualitatively showed more combinations of V-J pairings than vCAR or vIL7 (**Figure 5B**). Finally, we assessed T cell epitope spreading of the top 10 clones. While vIL7 groups showed the highest peak frequency, there was no consistency between groups (**Figure 5C**). When assessing the CD3 receptor nucleotide sequence between groups, qualitatively vIL7FL had a dense representation of sequences (**Figure 5D**). We observed similar trends for the TCR alpha chain (**Supplementary Figure 5**). While not significant, we did observe the potential for vIL7FL to alter T cell diversity.

## Discussion

CAR T cell therapy in GBM clinical trials has encountered significant challenges, including limited T cell trafficking to the tumor site, inadequate abundance, tumor antigen loss, tumor immunosuppression, and adverse effects (6, 73). While preclinical studies are making enormous strides in addressing these limitations, these models are mainly limited to immunocompromised/lymphodepleted mice and/or antigen homogenous glioma to achieve a therapeutic effect (40, 74–76). Here we utilized EGFRvIII heterogeneous glioma with non-lymphodepleting pre-conditioning to preserve the endogenous immune system and emulate antigen heterogeneity present in GBM. In our study, we demonstrated that IL7 expression in CAR T cells increased intratumoral CAR T cells, and co-expression with Flt3L enhanced cDC populations. IL7 and IL7 Flt3L CAR T cells, in combination with 0.5 Gy TBI, improved overall survival in EGFRvIII heterogeneous tumors.

We genetically modified third generation EGFRvIII CAR T cells to secrete mouse IL7 and/or Flt3L. Previous studies show the benefits of IL7 on T cell survival, proliferation, and memory T cell formation (18, 77, 78) and Flt3L on DC survival, differentiation, and expansion (44, 45). Similar to previous findings, IL7 signaling in CAR T cells enhanced cell proliferation *in vitro* (17, 28). When delivered in a 5 Gy TBI EGFRvIII homogenous tumor model, vIL7 and vIL7FL increased the proportion of intratumoral CD8 CAR T cells compared to conventional vCARs.

We assessed the efficacy of vIL7FL in a more rigorous model using 0.5 Gy TBI and 50% EGFRvIII positive and negative tumors. 0.5 Gy irradiation retains a proportion of leukocyte and splenic DC populations as opposed to 4 Gy irradiation, which severely depletes these cells (13). Delivery of vIL7 and vIL7FL significantly increased intratumoral CD8 CAR T cells. Notably, vIL7FL increased CD8 CAR T cells compared to vIL7 CAR T cells *in vivo* despite tending to have lower mean secretion of IL7 (not significant) *in vitro*. This suggests the possibility of a synergistic effect of IL7 and Flt3L secretion on CAR T cell survival. Flt3L has been shown to prevent the decline of CD28 and IFNγ secretion in CD8 T cells as well as reduce PD-L1 expression on DCs and macrophages (79). However, we didn’t observe significant differences in IFNγ secretion *in vitro*, PD1 expression on CAR T cells *in vivo*, or differential gene expression of PD-L1 (**Supplementary Figures 1, 3**). Interestingly, vIL7FL did not significantly enhance CD8 CAR T cells compared to vIL7 in the 5 Gy TBI model. Potentially, Flt3L is interacting with other immune cells in the 0.5 Gy model that were not present with 5 Gy TBI that promote CD8 CAR T cell survival. However, future studies of select populations would be necessary to determine this.

Additionally, we examined CAR- T cells because endogenous tumor infiltrating lymphocytes have the potential to recognize and kill autologous tumor cells (14–16). Delivering any CAR T cell significantly reduced the CAR- T cell exhaustion phenotype, furthermore vIL7 decreased exhaustion compared to vFL. While IL7 has been shown to reduce exhaustion and regulate metabolism in CAR T cells, we did not see any differences in the CAR+ T cell exhaustion phenotype, as exhaustion remained low in all T cell groups most likely due to the addition of the 4-1BB domain that has been shown to reduce exhaustion (**Supplementary Figure 3**) (31, 80). vIL7 enhanced absolute counts of CD8+CAR- CD69+ T cells compared to PBS, vCAR, and vFL. CD69 is surrogate marker of early T cell activation because it is rapidly produced after TCR engagement (81).

We evaluated the effect of vIL7FL on DC populations in the tumor-bearing hemisphere. In the steady-state mouse brain, subcutaneous Flt3L delivery has been shown to increase antigen-presenting cDC populations (82). Additionally, these cDCs arose from the differentiation of pre-DC progenitors that had migrated into the brain. Alternatively, Flt3L administration in rat brains has increased pDC populations (49). We elucidated that vIL7FL enhanced intratumoral cDCs including the migratory antigen-presenting population known as CD103+XCR1+ cDCs (83, 84).

Delivery of vIL7 or vIL7FL in an EGFRvIII heterogeneous 0.5 Gy model significantly enhanced overall survival. In our model, the effects of IL7 dominated despite DC infiltration from Flt3L co-expression. Potentially, the lack of proper DC maturation affected T cell priming. Poly(I:C) is a toll-like receptor 3 (TLR3) agonist that can promote type I interferon (IFN) signaling in DCs as well as activate CD8 T cells (85, 86). Future investigations could adjuvant CAR T cells with poly(I:C) to potentially enhance anti-tumor T cell responses. While in our study one vCAR and vFL animal did survive, we speculate that this could be due to the bystander effect or immunogenicity. A previous study showed preconditioning mesothelin tumors with up to 25% antigen-negative cells with a non-lymphodepleting dose of cyclophosphamide resulted in CAR T cells having a curative effect (61).

A limitation of this study is the utilization of a syngeneic mouse model compared to spontaneously occurring tumors. Transplantable tumor cell lines tend to be more immunogenic and elicit non-naturally occurring immune responses compared to genetically induced mouse models. We chose the CT2A mouse model due to its low immunogenicity and immunosuppressive microenvironment compared to GL261 and SMA-560 models (87, 88). However, CT2A tumors can be less correlated to patient immune phenotypes than GL261 tumors and can become immunologically active after surgical resection (89). In addition, forced expression of luciferase in tumor cells was necessary to distribute animals based on bioluminescent tumor load, however, luciferase can increase tumor immunogenicity (90). Thus, the efficacy of vIL7FL should be tested in other models. Another limitation is tumor antigen expression. Patients with EGFRvIII positive tumors show varying levels of EGFRvIII expression at different locations within the tumor (35–37). In our study, treating mice with 50% EGFRvIII-positive and -negative tumors with vIL7 or vIL7FL increased survival compared to conventional CAR T cells. However, we can anticipate that decreasing the EGFRvIII positive tumor cell ratio could limit efficacy. Therefore, it remains to be seen whether vIL7FL will have the same efficacy in naturally occurring tumors with varying levels of heterogeneity.

Overall, this data emphasizes the ability of IL7 expression to improve CAR T cell abundance in GBM. While vIL7FL increased intratumoral dendritic cells, future studies are necessary to determine the therapeutic impact of intratumoral cDCs in brain tumors. IL7 expressing CAR T cells improved overall survival in mice pre-treated with a non-lymphodepleting dose of irradiation – allowing for retention of host immune cells – thus, the use of IL7 expressing CAR T cells can open opportunities for combinations of other immunotherapies in glioblastoma.

## Data availability statement

The datasets presented in this study can be found in online repositories. The names of the repository/repositories and accession number(s) can be found below: GEO under accession ID: GSE215873. 

## Ethics statement

The animal study was reviewed and approved by Institutional Animal Care and Use Committee at Duke University.

## Author contributions

SLS: conception, design, methodology, data acquisition, data analysis, writing original manuscript. NM: design, methodology, data acquisition, data analysis, manuscript review. EI: methodology, data acquisition, data analysis SHS: methodology and data acquisition DSW: data acquisition ARA: data acquisition TS: resources, supervision, manuscript review LS: conception, design, methodology, manuscript review JHS: methodology, resources, manuscript review RVB: methodology, resources, supervision, investigation, funding acquisition, manuscript review. All authors contributed to the article and approved the submitted version.
