# Supplementary material for: IL7 and IL7 Flt3L co-expressing CAR T cells improve therapeutic efficacy in mouse EGFRvIII heterogeneous glioblastoma
Source: Front Immunol. 2023 Feb 3;14:1085547. doi: 10.3389/fimmu.2023.1085547 (PMC9936235; doi:10.3389/fimmu.2023.1085547)
Supplement: Supplementary file 1 [file DataSheet_1.docx]

Supplementary Material

# Supplementary Data

## IFNγ and Granzyme B secretion

**Supplementary Figure 1.** CAR T cells were made using activated mouse splenocytes and transduced on day 2. CAR T cells were alone or co-cultured with tumor cells (vIII, vIII+2A, or 2A) with an effector:target (E:T) ratio of 10:1. 24 hours later the supernatant was collected for IFNγ or Granzyme B ELISA. Experiments represent 3 biological replicates with mean and SEM plotted. Statistical analysis was conducted within each tumor group using a one-way ANOVA with a Tukey’s multiple comparison test.

## Gating strategy for T cell and dendritic cell phenotypes.


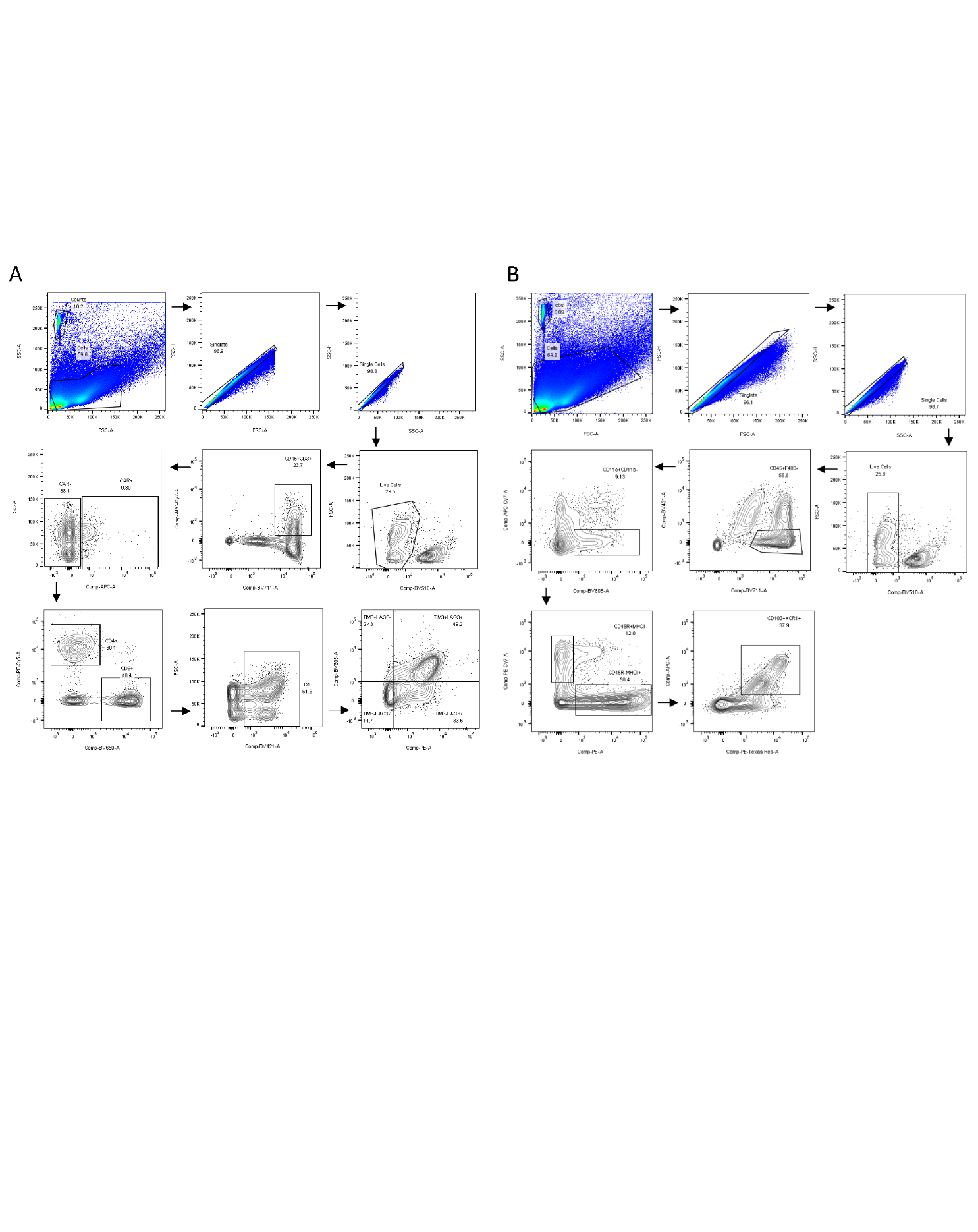


**Supplementary Figure 2.** Animals were inoculated with 50% vIII and 2A tumor cells and IVIS imaged 5 days later. On day 6, 0.5Gy TBI was applied and 2x10^6 CAR T cells were injected intracranially the following day. Flow cytometry was performed on the tumor-bearing hemisphere isolated on day 14 and split between two panels A) T cell panel B) DC panel

## Flow Cytometry analysis of CAR T cells delivered in 0.5 Gy pre-conditioned EGFRvIII heterogeneous model


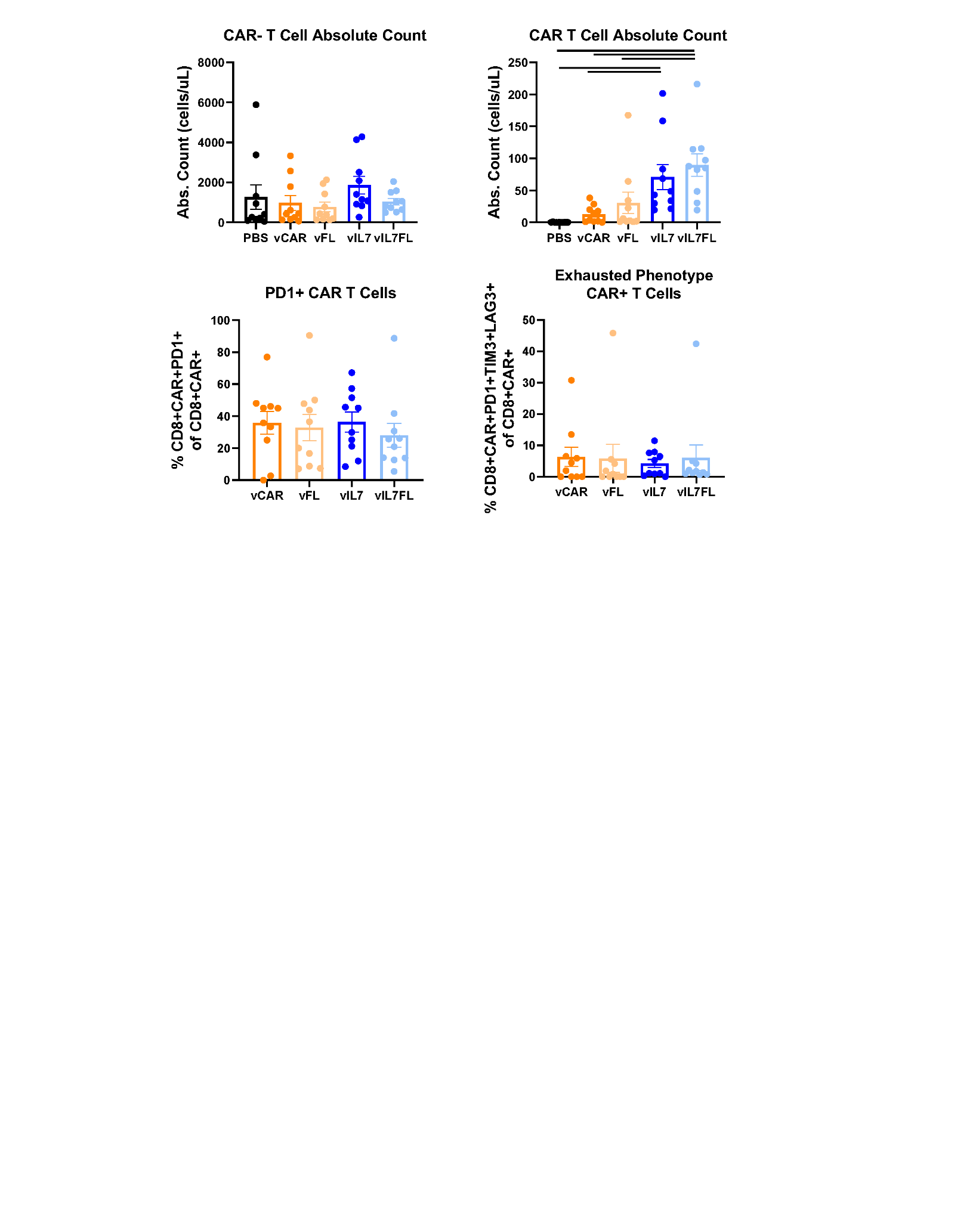


**Supplementary Figure 3.** Animals were inoculated with 50% vIII and 2A tumor cells and IVIS imaged 5 days later. On day 6, 0.5Gy TBI was applied and 2x10^6 CAR T cells were injected intracranially the following day. Flow cytometry was performed on the tumor-bearing hemisphere isolated on day 14 and split into two panels with the T cell panel shown. Statistical test was a one-way ANOVA with a Tukey’s multiple comparison test with mean and SEM plotted.

## 0.5 Gy EGFRvIII heterogeneous bioluminescent imaging and predicted cytokine signaling


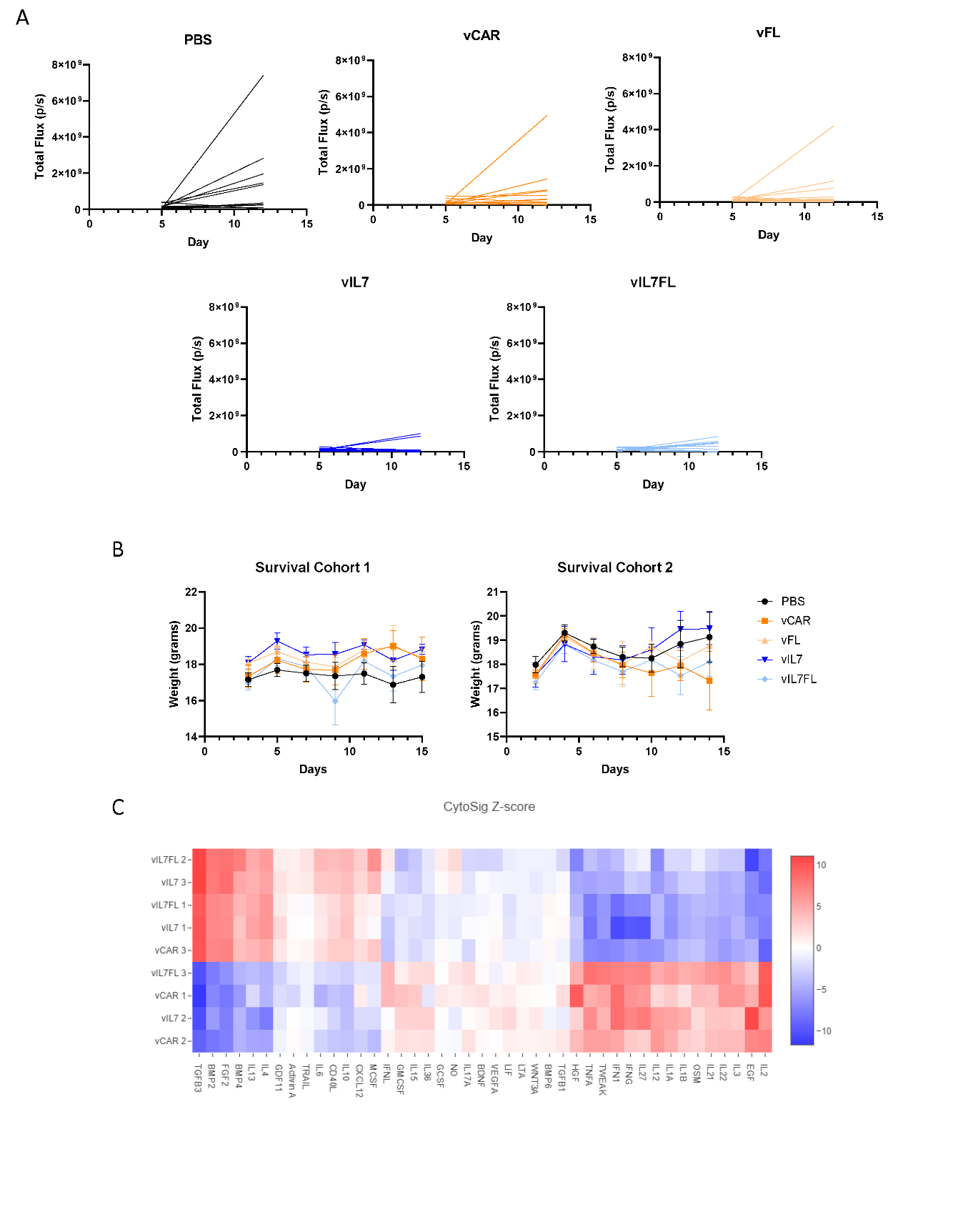


**Supplementary Figure 4.** Animals were inoculated with 50% vIII and 2A tumor cells and IVIS imaged 5 days later. On day 6, 0.5Gy TBI was applied and 2x10^6 CAR T cells were injected intracranially the following day. A) Bioluminescent imaging was performed on day 5 and day 12 from the animals shown in the survival study. The maximum total flux using time-lapse imaging was plotted. B) Animals weights days after CAR T cell delivery C) Bulk RNA sequencing was performed on RNA isolated from tumors on day 14. Predicted cytokine signaling was found with mean-centralized log-transformed data using the Cytosig platform

## T Cell receptor alpha chain diversity

**
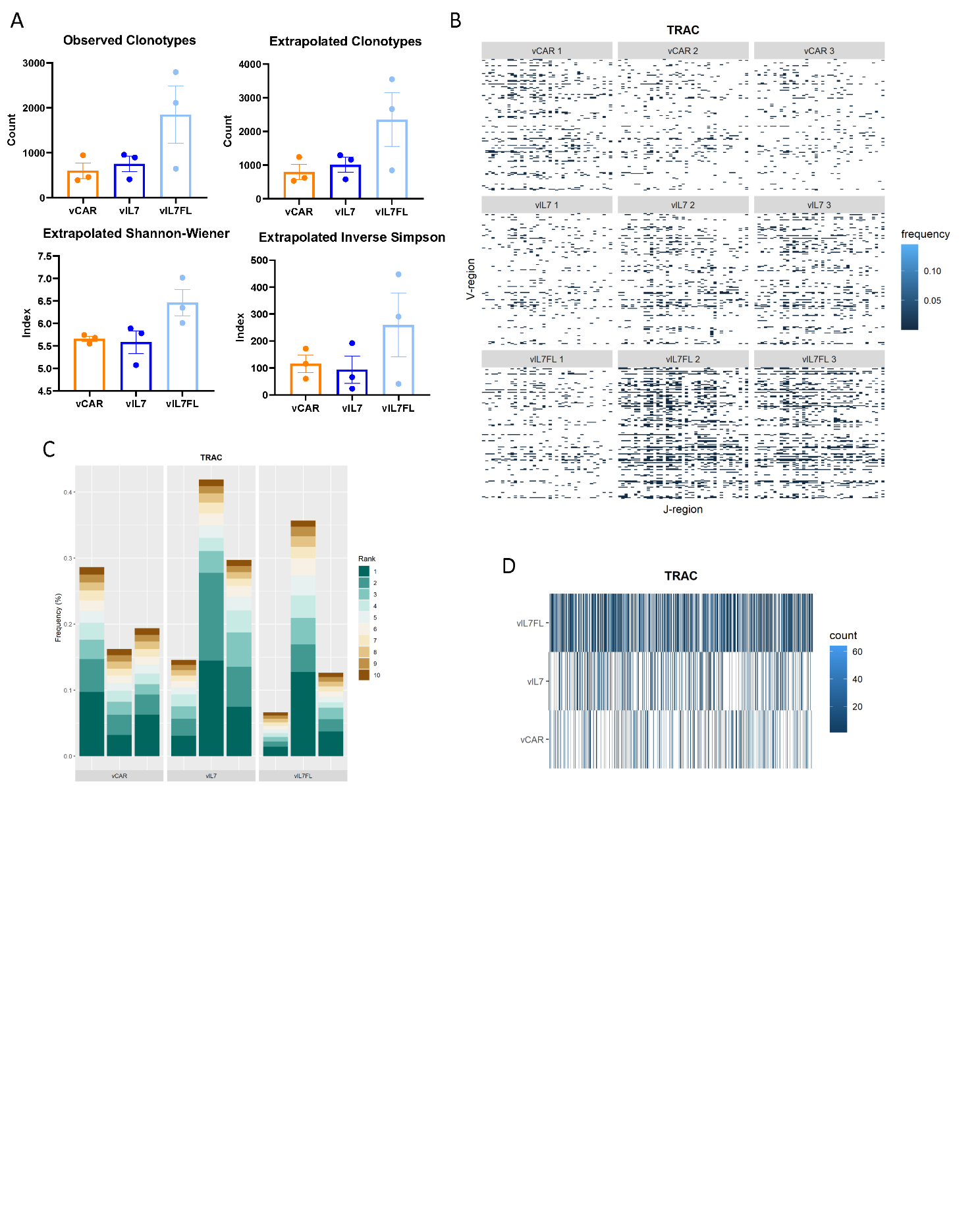
**

**Supplementary Figure 5.** Animals were inoculated with 50% vIII and 2A tumor cells and IVIS imaged 5 days later. On day 7, 0.5Gy TBI was applied and 2x10^6 CAR T cells were injected intracranially the following day. RNA was extracted from the tumor-bearing hemisphere and processed for immune repertoire screening A) Diversity metrics of TRAC B) Heat map of V and J pairings of TRAC C) The frequency of the top 10 clonotypes in TRAC. D) Visualization of TRAC clonotypes combing biological replicates. Statistical test was a one-way ANOVA with a Tukey’s multiple comparison test with mean and SEM plotted.
